# Berberine-induced autophagic cell death by elevating GRP78 levels in cancer cells

**DOI:** 10.18632/oncotarget.14959

**Published:** 2017-02-01

**Authors:** Xiaoqin La, Lichao Zhang, Zhuoyu Li, Peng Yang, Yingying Wang

**Affiliations:** ^1^ Institute of Biotechnology, Key Laboratory of Chemical Biology and Molecular Engineering of National Ministry of Education, Shanxi University, Taiyuan 030006, China; ^2^ School of Life Science, Shanxi University, Taiyuan 030006, China

**Keywords:** berberine, autophagy, cancer cell death, GRP78, VPS34

## Abstract

Berberine, an isoquinoline alkaloid extracted from *Coptidis Rhizoma*, has been shown to induce cancer cell autophagic death. Glucose regulated protein 78 (GRP78) is associated with stress-induced autophagy. However, the related mechanisms between berberine-induced cancer cell autophagy and GRP78 remain to be elucidated. Here, we report that berberine can induce autophagic cancer cell death by elevating levels of GRP78. These results further demonstrated that berberine enhanced GRP78 by suppression of ubiquitination / proteasomal degradation of GRP78 and activation of ATF6. Moreover, fluorescence spectrum assay revealed that berberine could bind to GRP78 and form complexes. Finally, co-IP analysis showed that the ability of GRP78 to bind to VPS34 was increased with berberine treatment. Taken together, our results suggest that berberine induces autophagic cancer cell death via enhancing GRP78 levels and the ability of GRP78 to bind to VPS34.

## INTRODUCTION

Autophagy is the cellular process that delivers damaged organelles and invasive bacteria to lysosomes for degradation in order to maintain intracellular homeostasis during various cell stresses [1]. These substrates are engulfed along with bulk cytoplasm by a double-membrane, with the extension and fusion of the membrane edge, resulting in the formation of the autophagosome [2]. After the fusion of the autophagosome and lysosome, the degradation products are recycled back into the cytosol and are reused to enhance cell survival during nutrient deprivation [3]. The entire autophagy process is complex and involves many critical proteins. Among these, LC3 (targeted to autophagosome membranes) [3] and class III phosphatidylinositol 3-kinase (also known as VPS34) are responsible for membrane remodeling and trafficking events in autophagy [4]. In addition, autophagy can be inhibited at different stages by various inhibitors, such as 3-MA and wortmannin, which block the initial phase of the autophagic process by specifically inhibiting PI3K activity. CQ and Bafilomycin A1 can suppress the fusion of the autophagosome and lysosome during later stages.

Accumulating evidence suggests that autophagy serves a largely cytoprotective role in physiologically relevant conditions. The cytoprotective function of autophagy is activated in many circumstances by suppressing apoptosis [5]. It is now being recognized that autophagy is not only a survival response to growth factor or nutrient deprivation, but also an important mechanism underlying tumor cell suicide [6]. Interminable autophagy has been shown to enhance anticancer drug-induced cell death. In contrast, disruption of autophagy promotes tumorigenesis [7]. Thus, induction of autophagy has been implicated as a cancer suppressive mechanism in drug-induced cell death [8]. Therefore, elevating the basal levels of autophagy may be a useful approach in cancer therapeutics.

One potential regulator of autophagy is activation of the unfolded protein response (UPR), an endoplasmic reticulum stress pathway [9, 10]. UPR activation occurs when unfolded proteins accumulate within the endoplasmic reticulum, resulting in the release of protein chaperone glucose-regulated protein 78 (GRP78, also known as BiP or HSPA5) from either PKR-like endoplasmic reticulum kinase (PERK, EIF2AK3), inositol requiring enzyme 1 (IRE1, ERN1), and/or activating transcription factor 6 (ATF6) [11]. GRP78, a key upstream activator of the UPR, participates in promoting protein folding, assembly and degradation, endoplasmic reticulum stress sensing, and cellular calcium homeostasis [12, 13]. High levels of GRP78 contribute to stress-induced autophagy, and functional blockade of the proteasome induces GRP78 to promote autophagosome formation [12, 14–19].

As a major active compound of Coptidis Rhizoma (Huanglian in Chinese), berberine is an isoquinoline alkaloid, which has long been used as a nonprescription oral drug in China for the treatment of gut infections and diarrhea [20]. Historically, berberine has been developed as a clinical drug due to its diverse efficacy, as an antimicrobial, antihyperlipidemic, antidiabetic, anti-inflammation and antioxidant factor [21, 22]. Importantly, the antineoplastic activities of berberine have been intensive studied and reported since the 1990s [20]. A large number of studies have revealed the cytotoxicity, metastasis inhibition and antiangiogenic properties of berberine [23–28]. Other studies have focused on cell-cycle checkpoint control, DNA repair and regulation of initiating oxidative stress in cancer cells, which highlight new antitumor mechanisms of berberine to light.

Recent studies have revealed that berberine can trigger autophagic cell death of tumor [8], and its beneficial effects are receded when the autophagic process is genetically or pharmacologically inactivated, suggesting that autophagy is indispensable for the protective effects of berberine [8, 29]. Here, we revealed that berberine could elevate GRP78 to induce autophagy in cancer cells, which may represent a promising candidate for cancer therapy.

## RESULTS

### Berberine treatment induced cell death in some cancer cell lines

**Figure 1:**
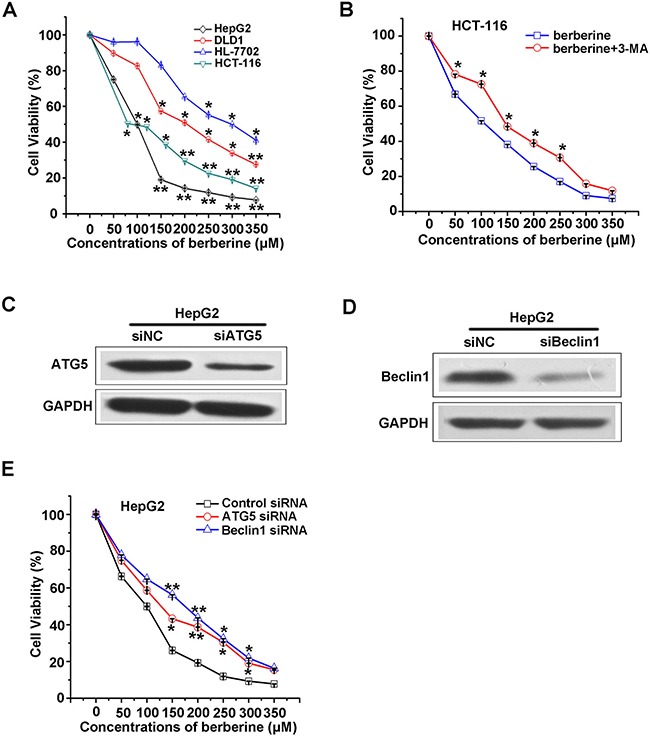
Berberine treatment induced autophagic cancer cells death **A**. HCT-116, DLD1, HepG2 and HL-7702 cells were treated with different concentrations of berberine for 24 h. Cell viability was detected using the MTT assay and plotted against berberine concentrations, n=3. The cell viability curve was fitted using the Hill equation. IC_50_ indicated the concentration at which 50% of the cells survived. **B**. Viability of HCT-116 cells after treatment with berberine plus or minus 3-MA (10mM) was measured by MTT. **C**. Expression of ATG5 in HepG2 cells transfected with control or ATG5 siRNA was detected by western blot. **D**. Expression of Beclin1 in HepG2 cells transfected with control or Beclin1 siRNA by western blot are shown. **E**. The cytotoxicity of berberine can be attenuated by introducing siRNA against ATG5 and Beclin1 into HepG2 cells. All experiments were performed in triplicate and the results were analyzed for statistical significance (*p<0.05, **p<0.01).

Previous studies have confirmed that berberine can inhibit the growth of cancer cells with a relatively low IC_50_ *in vitro* [23–27, 30–34]. To test the toxicity of berberine in colon cancer cells HCT-116, DLD1 and hepatic carcinoma cells HepG2, normal liver cell line HL-7702, the cell viability corresponding to a series of berberine concentrations was measured (Figure 1A). These results showed that DLD1 treated with 100 μM berberine caused a significant decrease in cell viability. HCT-116 and HepG2 were more sensitive to berberine treatment, in which 50 μM produced an obvious inhibition effect. Interestingly, in the normal liver cell line HL-7702, there was a slight decrease in viability when the berberine concentration was less than 150 μM, suggesting that berberine was more effective in cancer cells compared to normal cells. The cell viabilities were plotted against berberine concentrations, revealing that the IC_50_ of berberine was 80 μM, 100 μM, 200 μM and 300 μM in HCT-116, HepG2, DLD1 and HL-7702, respectively. The addition of 3-MA reduced the effect of berberine on HCT-116 cell viability (Figure 1B). Consistent with these findings, silencing of ATG5 and Beclin1 attenuated berberine-induced HepG2 cell death (Figure 1C, 1D and 1E), indicating that induced autophagy may function as one anti-cancer mechanisms of berberine.

### Berberine activated autophagy in HCT-116 cells

**Figure 2:**
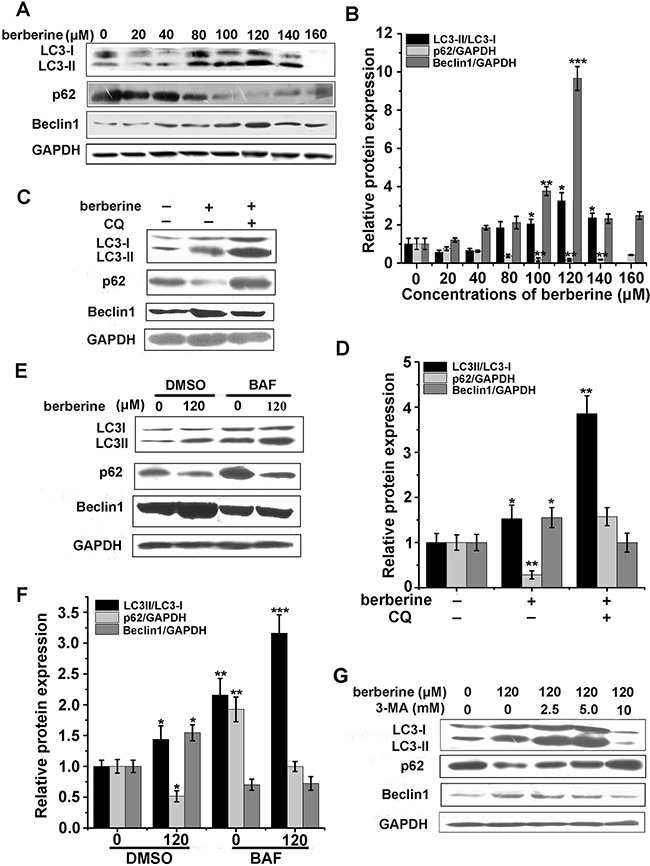
Berberine-activated autophagy in HCT-116 cells **A** and **B**. Western blots of LC3, p62, Beclin1 and GAPDH were performed on HCT-116 cell lysates treated with berberine at the indicated concentrations for 24 h. The relative protein expression was calculated by Image J. **C-D**. HCT-116 cells were treated with the indicated concentrations of berberine for 24 h with or without CQ (50 μM). The levels of LC3, p62 and Beclin1 were monitored by western blot of the cell lysates (C) and the relative protein expression was calculated by Image J (D). **E-F**. Western blotting analysis of LC3-II/LC3-I, p62 and Beclin1 levels (E) and the relative protein expression were quantified by Image J (F) in HCT-116 cells treated with berberine at the indicated concentrations for 24 h, in the absence or presence of 37.5 μM BAF (treated in combination with berberine). **G**. Protein expression levels of LC3, p62 and Beclin1 were analyzed by western blot in HCT-116 cells after treatement with berberine at the indicated concentrations for 24 h, in the absence or presence of 3-MA at different concentrations (treated in combination with berberine). Three independent experiments were performed, and the data were expressed as the mean ± SD. *p<0.05, **p<0.01, ***p<0.001 were compared to the untreated group.

To determine whether berberine treatment resulted in autophagic cell death, the expression levels of LC3-II, p62 and Beclin1, indicators of autophagy, were investigated in HCT-116 cells. These data showed that the expression of LC3 and Beclin1 were significantly increased with berberine treatment for 24 h, while the levels of p62 were reduced in a dose-dependent manner, peaking at 120 μM (Figure 2A and 2B). Because the accumulation of LC3-II may be attributed to an increase in autophagosome formation or decrease in lysosomal fusion and degradation, we next used chloroquine (CQ) and Bafilomycin A1 (BAF), inhibitors of the autophagosome, to block autophagic flux. These results showed that CQ or BAF treatment resulted in further accumulation of LC3-II in HCT-116 cells treated with berberine (Figure 2C, 2D, 2E and 2F), which exclude the possibility of lysosomal dysfunction caused LC3-II accumulation.

**Figure 3:**
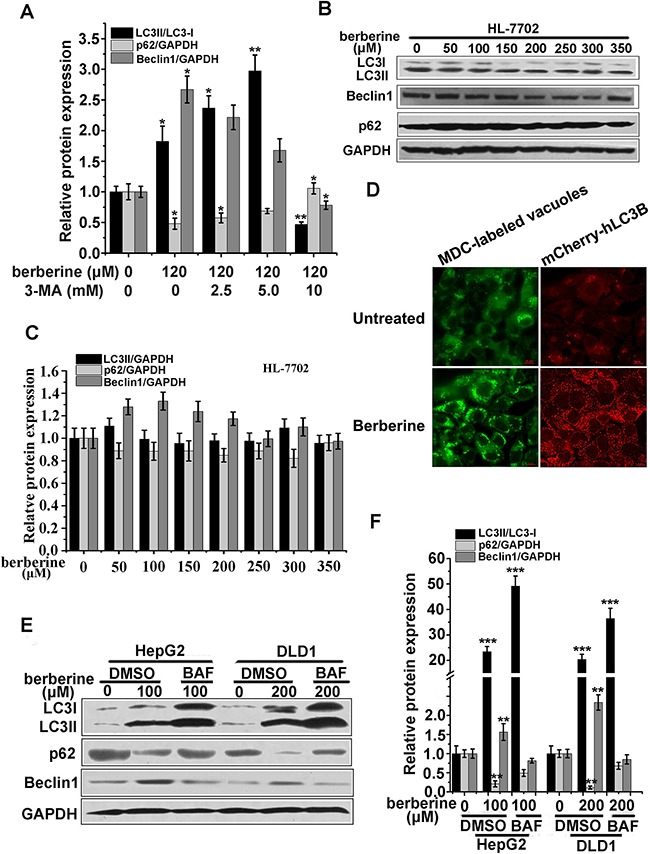
Berberine activated autophagy in cancer cell lines of HCT-116, DLD1, and HepG2 **A.** Quantification of the relative protein expression is shown in Figure 2G of three independent experiments using Image J software. **B** and **C.** Western blot analysis of LC3, Beclin1 and p62 expression (B) and the relative protein expression were evaluated by Image J (C) in HL-7702 cells treated with various concentrations of berberine. **D.** HCT-116 cells incubated with 0.05 mM monodansylcadaverine (MDC) for 10 min after treatment with the indicated concentration of berberine 24 h (left panel) or transfected with mCherry-hLC3B treated with 120 μM berberine for 24 h (right panel). The cells were then analyzed using fluorescence microscopy. Scale bar, 5 μm. **E-F.** HepG2 and DLD1 cells were treated with the indicated concentrations of berberine for 24 h with or without BAF co-incubation. LC3, Beclin1 and p62 were analyzed by western blotting analyses (E) and relative protein expression levels were calculated by Image J (F). Three independent experiments were performed, and the data were expressed as the mean ± SD. *p<0.05, **p<0.01, ***p<0.001 compared to the untreated group.

Furthermore, 3-MA, an inhibitor of autophagosome formation, was applied to block autophagic flux. These results showed that 10 mM 3-MA could inhibit LC3-II expression levels (Figure 2G and 3A). In contrast, the expression of LC3-II produced no obvious changes in the protein levels when treated with a series of berberine concentrations in normal hepatocytes HL-7702 (Figure 3B and 3C).

### Confirmation of autophagy induced by berberine treatment

**Figure 4:**
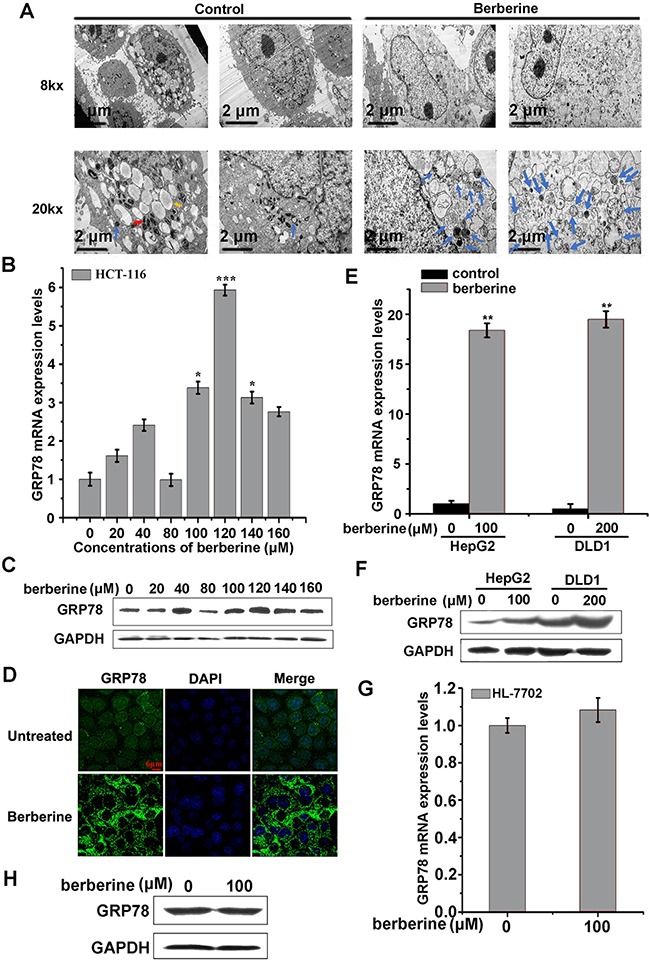
GRP78 was elevated in berberine treated cancer cell lines **A.** Electron microscopic images of HepG2 control and berberine-treated cells (120 μM) for 24 h. Lipid droplets (yellow arrow); autophagosomes and autolysosome of double-membrane, single-membrane, and multivesicular body-like vesicles (blue arrows); mitochondria (red arrow). Scale bars are indicated at the bottom. **B** and **C.** HCT-116 cells were exposed to 20, 40, 80,100,120,140 and 160 μM berberine for 24 h. GRP78 was detected by qRT-PCR (B) and western blotting analyses (C). **D.** HCT-116 cells were exposed to 120 μM berberine for 24 h, and the cells were then fixed and stained with GRP78-specific antibody and examined using fluorescence microscopy. **E** and **F.** HepG2 and DLD1 cells were exposed to the indicated concentrations of berberine for 24 h, and GRP78 was detected by qRT-PCR (E) and western blotting analyses (F). **G** and **H.** HL-7702 cells were exposed to 100 μM berberine for 24 h, and GRP78 was detected by qRT-PCR (G) and western blotting analyses (H).

Next, autophagic vacuole organelle (AVO) formation was detected and measured by staining with MDC. Berberine-treated HCT-116 cells showed stronger fluorescence intensity and a greater number of MDC-labeled particles compared with the control group (Figure 3D), indicating that berberine increased the formation of autophagosomes in the cytoplasm. Similar to MDC staining, exogenous mCherry-hLC3B was overexpressed and assembled into autophagosomes upon berberine treatment (Figure 3D). Similar results were obtained in DLD1 and HepG2 cells treated with berberine (Figure 3E and 3F). Importantly, transmission electron microscopy observations showed that in berberine-treated HepG2 cells, autophagosome-related structures were observed (Figure 4A), which was not as easily visible in control cells. These data suggested that berberine could strongly promote cellular autophagy in HCT-116, DLD1, and HepG2 cells, but not in normal hepatocytes HL-7702 cells.

### Berberine treatment enhanced GRP78 expression levels

Previous studies have demonstrated that GRP78 was an autophagy inducer under conditions of glucose starvation [35]. We further explored whether GRP78 was involved in berberine-induced autophagy. First, HCT-116 cells were treated with a gradient of berberine for 24 h, and the expression of GRP78 was then measured. These results showed that cell autophagy was accompanied with an increase in GRP78 expression (Figure 4B and 4C). Furthermore, immunofluorescence results showed that the fluorescence intensity of GRP78 was strengthened with berberine treatment (Figure 4D). In addition, we observed an enhancement of GRP78 in DLD1 and HepG2 cells (Figure 4E and 4F). Importantly, GRP78 showed no obvious changes in non-neoplastic HL-7702 cells with berberine treatment (Figure 4G and 4H). Taken together, these results suggested that berberine induced cancer cell autophagy, which was accompanied by an increase in GRP78 that was not observed in normal cells.

### GRP78 played a dominant role in the modulation of autophagy

**Figure 5:**
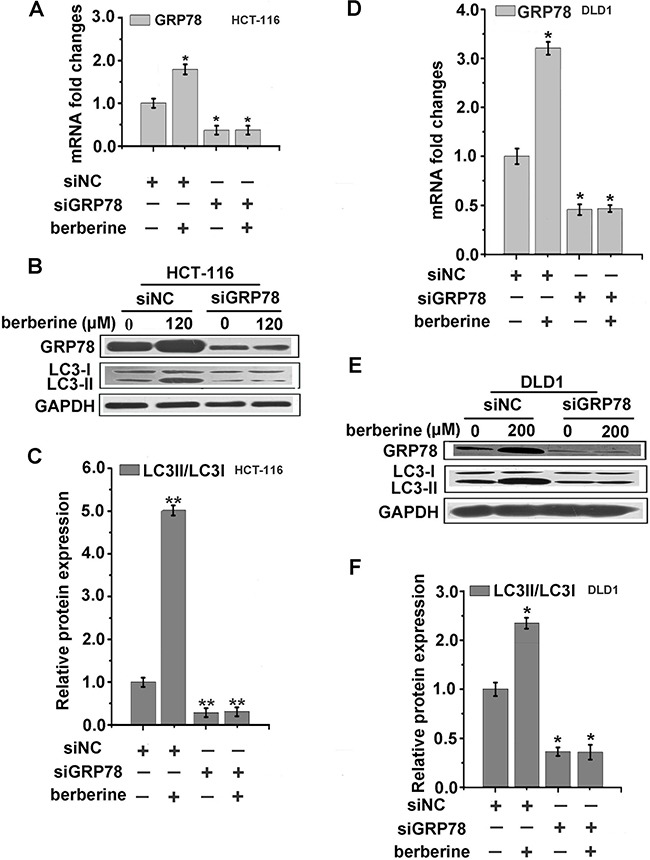
Knockdown of GRP78 abolished the autophagy effect of berberine in HCT-116 and DLD1 cells **A-F.** HCT-116 and DLD1 cells were transfected with control siRNA or siRNA targeting GRP78, respectively. After 48 h, the cells were treated with indicated concentrations of berberine for 24 h, then GRP78 was measured by qRT-PCR (A and C) and GRP78, LC3 were examined by western blotting analyses (B and E). The LC3-II/LC3-I relative protein expression levels were calculated by Image J (C and F). Data were expressed as the mean ± SEM of three different experiments. *p<0.05 and **p<0.01 vs. respective control.

**Figure 6:**
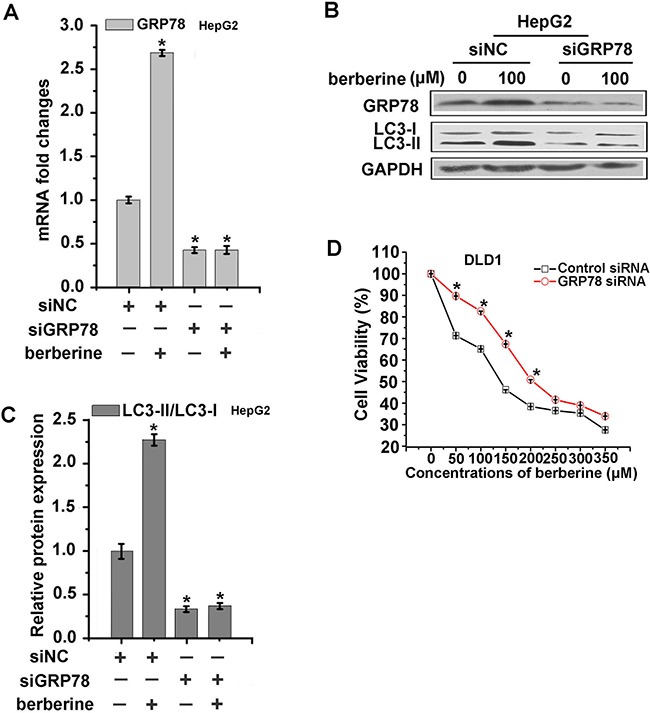
GRP78 played a dominant role in the modulation of autophagy in HepG2 and DLD1 cells **A-C.** HepG2 cells were transfected with control siRNA or siRNA targeting GRP78, respectively. After 48 h, the cells were treated with indicated concentrations of berberine for 24 h, then GRP78 was measured by qRT-PCR (A) and GRP78, LC3 were examined by western blotting analyses (B). The LC3-II/LC3-I relative protein expression levels were calculated by Image J (C). **D.** The cytotoxicity of berberine can be attenuated by introducing siRNA against GRP78 into DLD1 cells. Data were expressed as the mean ± SEM of three different experiments. *p<0.05 vs. respective control.

To further assess whether berberine-induced GRP78 expression is correlated with autophagic cancer cell death, we transfected HCT-116, DLD1, HepG2 cells with GRP78 siRNA. The cells were then treated with berberine at the optimum concentration for 24 h. The protein expression of LC3 was then examined. These results showed that three types of cancer cells treated with 10 nM siRNA for 48 h could lead to approximately 50% repression of GRP78 expression compared to control (Figure 5A, 5D and 6A), and the expression of LC3 were significantly decreased in the three types of cancer cell lines depleted of GRP78 cells (Figure 5B, 5C, 5E, 5F, 6B and 6C). Furthermore, the siRNA of GRP78 was utilized to detect cell viability, and MTT results indicated that DLD1-siGRP78 cells exhibited greater resistance to berberine treatment (Figure 6D). Taken together, these data suggested that GRP78 played a critical role in berberine-induced autophagic cancer cell death.

### Berberine elevated GRP78 expression via increased ATF6 levels

**Figure 7:**
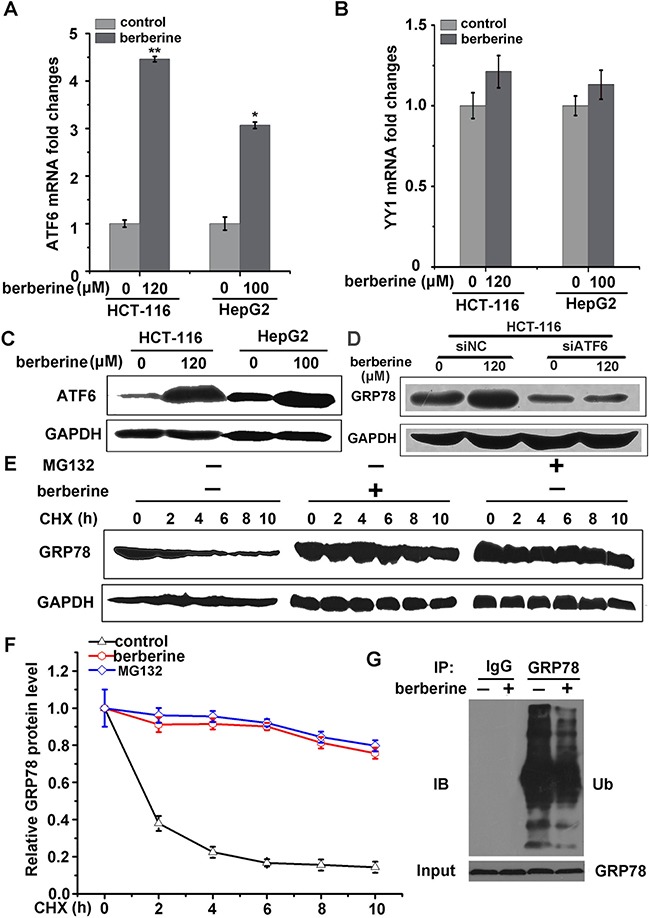
Berberine induced GRP78 expression by increased ATF6 expression and GRP78 stability **A.** HCT-116 and HepG2 cells were treated with berberine for 24 h. ATF6 mRNA was measured by qRT-PCR. **B.** HCT-116 and HepG2 cells were treated with berberine for 24 h. YY1 mRNA was measured by qRT-PCR. **C.** Western bloting analyses were performed to examine ATF6 expression levels in HCT-116 and HepG2 cells. **D.** HCT-116 cells were transfected with control siRNA or siRNA targeting ATF6. After 48 h, the cells were treated with the indicated concentrations of berberine for 24 h, and GRP78 was measured by western blot. **E.** In the presence of berberine or proteasome inhibitor MG132 (5 μM), HCT-116 cells were treated with cycloheximide (CHX, 0.1 mg/ml) for the indicated times and GRP78 expression was then analyzed by western blot. **F.** Quantification of GRP78 expression was performed on three independent experiments using Image J software and normalized against vehicle control (Figure 6E). **G.** HCT-116 cells were treated with MG132 in the presence or absence of berberine for 24 h. GRP78 was immunoprecipitated, and GRP78 ubiquitination was examined. Three independent experiments were performed, and the data were expresses as the mean ± SD. *p<0.05, **p<0.01 were compared to the untreated group.

It is known that ATF6 (activating transcription factor 6) and YY1 (Yin Yang 1) are the main transcription factors regulating GRP78 expression [11, 36]. To determine which transcription factor is required for berberine-induced GRP78, the expression of ATF6 and YY-1 was analyzed in HCT-116 and HepG2 cells using RT-PCR. Compared to control, the mRNA of ATF6 increased significantly in cells treated with berberine (Figure 7A), but YY1 expresses only showed a slight change (Figure 7B). Western blotting analysis of ATF6 after berberine treatment confirmed the increased expression (Figure 7C). Consistent with these findings, silencing of ATF6 largely attenuated berberine-induced GRP78 expression (Figure 7D). These data indicated a novel function of berberine on the ATF6-GRP78 cascade.

### Berberine inhibits the ubiquitination and proteasomal degradation of GRP78

To determine whether the half-life of GRP78 protein was increased by berberine treatment, HCT-116 cells were treated with the protein synthesis inhibitor cycloheximide at various time points. These results showed that GRP78 was degraded much slower in berberine-treated cells, similar to findings obtained in 26S proteasome inhibitor MG132-treated cells, suggesting that berberine-induced GRP78 elevation occurred via the ubiquitination proteasome system (Figure 7E and 7F). In addition, co-immunoprecipitation with an anti-GRP78 antibody was performed using an anti-ubiquitin antibody and revealed that the ubiquitination signals of GRP78 were attenuated after treatment with berberine (Figure 7G). These results suggested that berberine decreased GRP78 ubiquitination, further resulting in the suppression of proteasomal degradation andelevating the stability of GRP78.

### Berberine can interact with GRP78

**Figure 8:**
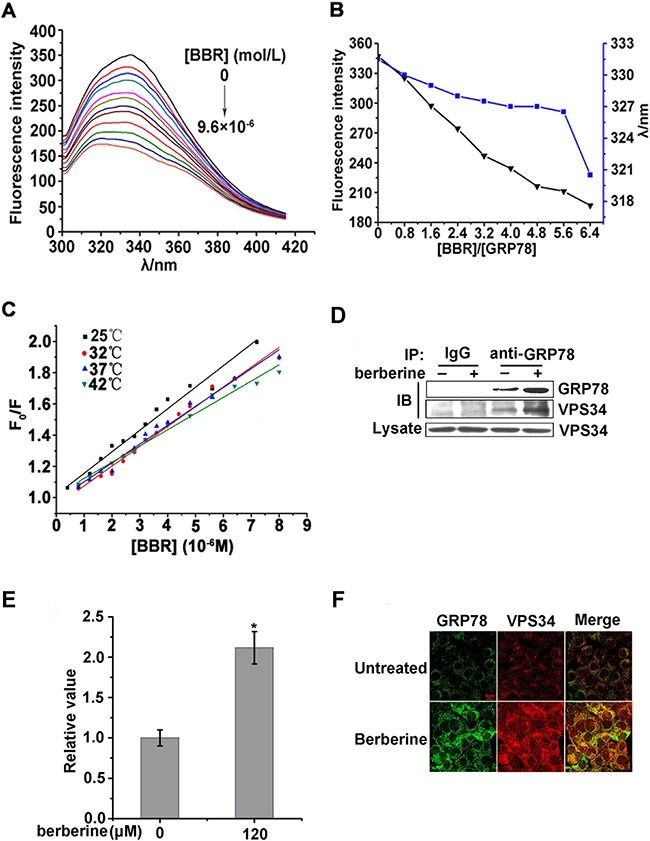
Berberine can interact with GRP78 and induce autophagy by facilitating the ability of GRP78 to bind to VPS34 **A.** Effect of berberine on fluorescence spectra of GRP78. λex = 295 nm, [GRP78] = 1.0×10^−6^ M, curves 1–12 [berberine]/[GRP78] = 0, 0.8, 1.2, 1.6, 2.4, 2.8, 3.2, 3.6, 4.8, 6.4, 8.0, 9.6, pH 7.4, at 42°C. **B.** The dots represent [berberine]/[GRP78] = 0, 0.8, 1.6, 2.4, 3.2, 4.0, 4.8, 5.6, 6.4, pH 7.4, at 42 °C. The fluorescence intensity of GRP78 declined gradually, and the blue-shift occurred for the emission maximum (from 331.5 to 320.5 nm). **C.** The values of Ksv, Kq and R^2^ were determined with Stern–Volmer equation by linear regression of plots of F_0_/F vs [Q] at different temperature. The Stern–Volmer equation: F_0_/F=1+Ksv[Q]=1+Kqτ_0_[Q], where F_0_ and F were fluorescence intensities of GRP78 before and after the addition of berberine, Kq was the quenching rate constant, and τ_0_ was the average lifetime of biomolecule without quencher that was valued at approximately 10^−8^s. [Q] was the concentration of berberine as a quencher, and Ksv was the Stern–Volmer quenching constant. **D.** HCT-116 cells were treated with berberine for 24 h. GRP78 was immunoprecipitated and IgG served as a negative control, VPS34 was then examined. **E.** Quantification of GRP78-VPS34 levels in the immunoprecipitated complex are shown in Figure 7D of three independent experiments using Image J software and normalized against the vehicle control. **F.** Confocal microscopy detection of co-localization of endogenous GRP78 with VPS34 in HCT-116 cells treated with berberine for 24 h. Permeabilized cells were stained with anti-GRP78 and anti-VPS34 antibody to detect GRP78 (green) (left) and VPS34 (red) (right). The merged images showed co-localizations (yellow) of GRP78 and VPS34 detected at multiple corresponding sites. Scale bars represent 6 μm.

To examine the mechanism underlying berberine inhibition of ubiquitination and proteasomal degradation of GRP78, His tagged-GRP78 was expresses and purified using the *E. coli* system (Supplementary Figure 1A, 1B and 1C). Fluorescence spectrum studies were performed to determine its ability to bind to berberine. These studies indicated that the fluorescence intensity of GRP78 gradually declined with an increasing concentration of berberine (Figure 8A), and a blue-shift was observed for the maximum emission (Figure 8B). These findings suggested that berberine could quench the fluorescence of GRP78, and the chromophore of Trp residue was placed in a more hydrophobic environment after the addition of berberine. Furthermore, the Stern–Volmer equation revealed that the quenching was not caused by dynamic collision but was due to static quenching caused by the formation of a complex between GRP78 and berberine (Figure 8C and Table 1).

**Table 1:** Stern-Volmer quenching constant of the interaction between berberine and GRP78

T(°C)	K_sv_/10^5^(L·mol^−1^)	K_q_/10^13^(L·mol^−1^·s^−1^)	R^2^
25	1.377	1.377	0.9737
32	1.265	1.265	0.9818
37	1.206	1.206	0.9858
42	1.038	1.038	0.9859

The values of Ksv, Kq and R^2^ were determined with Stern–Volmer equation: F_0_/F=1+Ksv [Q] =1+Kq τ_0_ [Q] by linear regression of plots of F_0_/F vs [Q] at different temperature.

### Berberine induced autophagy by facilitating the ability of GRP78 to bind to VPS34

GRP78 can form a complex with VPS34, which plays an important role in autophagosome formation [35, 37]. Next, we examined the ability of GRP78 to bind to VPS34 in HCT-116 cells treated with berberine. These results indicated that there was enhanced binding between GRP78 and VPS34 upon berberine treatment (Figure 8D and 8E). Moreover, immunofluorescence staining results showed that stronger co-localization of endogenous GRP78 and VPS34 after berberine treatment 24 h (Figure 8F).

## DISCUSSION

An increased number of studies have revealed that berberine can induce autophagic cell death by up regulating P38 MAPK in cancer cells [8]. In our study, we found that berberine could up-regulate GRP78, and the expression of ATF6 was positively correlated with GRP78 (Figure 7A, 7C and 7D). Thus, ATF6 may represent a new target of berberine, which is responsible for berberine-induced GRP78 elevation.

GRP78 can be degraded by the ubiquitination and proteasomal pathway via GP78 (E3 ubiquitin ligase) [38]. In our study, the ubiquitination of GRP78 was significantly decreased in the presence of berberine (Figure 7G). According to prior data, GRP78 acetylation at K353 prevents the ubiquitination and degradation of GRP78 [38]. Thus, we hypothesized that berberine may inhibit GRP78 ubiquitination by promoting GRP78 acetylation.

However, berberine may suppress ubiquitin-proteasome degradation by directly binding with GRP78. We performed a fluorescence spectrum assay, which revealed that berberine strongly bound to GRP78 and only one binding site was detected in GRP78 with berberine at 37°C (Supplementary Figure 2A and Supplementary Table 2). Moreover, thermodynamics parameters and the nature of the binding forces revealed that hydrogen bonding and vander Waals forces played a dominant role in the binding of berberine to GRP78 (Supplementary Table 3). However, the precise interaction site of berberine-GRP78 requires further detailed studies.

Although most evidence supports the role of autophagy in sustaining cell survival, cell death resulting from excessive cellular consumption has been attributed to unrestrained autophagy [39, 40]. Thus, there must be a threshold value for autophagy. If the level of cell autophagy exceeds the value, then cell death occurs. However, the value and factors that regulate the level of cell autophagy are still unknown. The expression of GRP78 in cancer cells was higher compared to normal cells, and GRP78 binds to VPS34 to promote autophagy. This study showed that berberine could enhance GRP78 levels and the ability of GRP78 to bind to VPS34. Thus, autophagy in cancer cells may reach the threshold value more readily compared to normal cells upon berberine treatment. Taken together, these explanations may account for the opposing results between cancer cells and normal cells observed with berberine treatment, and reveal that GRP78 is an important regulator of the autophagic paradox.

Moreover, previous studies have indicated that AMPK is a major intermediate in facilitating the beneficial effects of berberine [41–43]. Indeed, we observed increased p-AMPK^T183/172^ in berberine-treated HCT-116 and DLD1 cells, and siGRP78 largely decreased berberine-induced p-AMPK levels (Supplementary Figure 2B, 2C and 2D), indicating that AMPK may function as a downstream regulator of GRP78. Thus, our study provides a link between AMPK and GRP78 in berberine-induced cancer cell autophagic death.

In summary, our studies demonstrated that berberine could induce GRP78 expression by activation of ATF6 cleavage, and inhibition of ubiquitination and proteasomal degradation, further resulting in autophagy and cancer cell death.

## MATERIALS AND METHODS

### Reagents and antibodies

Berberine was purchased from J&K SCIENTIFIC LTD (China). The RPMI-1640 medium, Dulbecco,s modified Eagle,s (DMEM) medium, DMEM / F-12 1:1 medium and fetal bovine serum (FBS) were obtained from GIBCO (Grand Island, NY). MTT and monodansylcadaverin (MDC) were obtained from Sigma (St. Louis, MO). Trizol, PrimeScript RT Master Mix and SYBR green PCR master mix were from Takara (Shiga, Japan). GRP78 antibody was obtained from Abcam (Cambridge, UK). Antibodies for LC3, p62, AMPK, p-AMPK^T183/172^ and ATF6 were obtained from Proteintech (Chicago, USA). GAPDH, GRP78, Ub, Beclin1 and chloroquine were purchased from Bioworld Technology (Minneapolis, MN). VPS34 antibody was obtained from Cell Signaling Technology (Danvers, MA). FITC- and TRITC- secondary antibodies were obtained from Invitrogen (Carlsbad, CA). Bafilomycin and 3-methyladenine of autophagy inhibitors were purchased from Cayman Chemical (MI, USA). MG-132 (proteasome inhibitor) and cycloheximide (protein synthesis inhibitor) were obtained from Sigma (St. Louis, USA). Sodium dodecylsulfate polyacrylamide gel electrophoresis (SDS-PAGE) protein standard was obtained from Thermo Scientific. BCA protein assay kit was purchased from Beyotime.

### Cell culture and proliferation assay

Human colon carcinoma HCT-116 and DLD1, human hepatoma HepG2, human liver normal HL-7702 cell lines were obtained from the American Type Culture Collection and cultured in RPMI-1640 or DMEM / F12 1:1 medium supplemented with 10% FBS at 37 °C in a humidified tissue culture incubator containing 5% CO_2_.

Patient tumor cells and normal cells viabilities were measured using the MTT dye absorbance. Next, 5×10^3^ cells were plated in 96-wells and incubated in media, and then treated with drugs and incubated for 24 h. MTT was added into each well after 24 h, incubated for 4 h and then removed. Next, 100 μl DMSO was added, and the absorbance was determined using a BMG Labtech FLUOSTAR OPTIMA plate reader at 570 nm wavelength.

### RNA extraction, reverse transcription and real-time PCR analysis

Total RNA was extracted using Trizol reagent and reverse-transcribed into complementary DNA (cDNA) using 500 ng RNA for qPCR according to the manufacturer's instructions. qPCR was performed using the SYBR Green PCR Master Mix on an Applied Biosystems StepOnePlus Real-time PCR System (Applied Biosystems, Inc., Foster City, CA, USA). GAPDH served as an endogenous control. The relative expression of each targeted gene was normalized by subtracting the corresponding GAPDH threshold cycle (Ct) values using the ΔΔCt method. The primers used in this study are listed in Supplementary Table 1.

### RNA interference

Small interfering RNA (siRNA) targeting GRP78, ATG5, Beclin1, ATF6 and siGenome non-targeting siRNA were used for GRP78, ATG5, Beclin1, ATF6 knockdown and control, respectively. The set of siRNAs against GRP78 (sense 5′-AAGGUUACCCAUGCAGUUGTT-3′, antisense 5′-CAACUGCAUGGGUAACCUUTT-3′), the set of siRNAs against ATG5 (sense 5′-GACGUU GGUAACUGACAAATT-3′, antisense 5′-UUUGUCAG UUACCAACGUCTT-3′), the set of siRNAs against Beclin1 (sense 5′-CUGGACACGAGUUUCAAGATT-3′, antisense 5′-UCUUGAAACUCGUGUCCAGTT-3′), the set of siRNAs against ATF6 (sense 5′-GGAGACAGC AACGUAUGAUTT-3′, antisense 5′-AUCAUACGUU GCUGUCUCCTT-3′) and a negative control siRNA (sense 5′-UUCUCCGAACGUGUCACGUTT-3′, antisense 5′-ACGUGACACGUUCGGAGAATT-3′) were obtained from GenePharma. Briefly, cells were grown in 6-well plates and transiently transfected at 60-80% confluence with siRNA at a final concentration of 5 nM using siRNA-Mate™ transfection reagent (GenePharma) according to the manufacturer,s instructions. After 48 h transfection, the cells were incubated with fresh medium alone or with the indicated concentrations of berberine. After 24 h, the cells were harvested for GRP78 analysis of mRNA and protein levels by qPCR and western blotting analyses, respectively.

### Transfection assays, autophagosome detection and immunofluorescence analysis

To assess the alteration and location of targets, HCT-116 cells were plated on 12-well glass slides and grown to 60 to 80% confluence. A total of 2 μg mCherry-hLC3B plasmid was transfected using TurboFect transfection reagent (Thermo). Approximately 48 h later, cells were treated again with the indicated concentrations of berberine for an additional 24 h. The cells were then fixed in 4% paraformaldehyde in PBS for 30 min, and permeabilized with 0.3% Triton X-100 in PBS for 10 min fot cytoplasmic protein staining. Next, the cells were labeled with 0.05 mM MDC in PBS at 37°C for 10 min. The cells were then incubated with 4-6-diamidino-2-phenylindole (DAPI, Vector Lab) to visualize the nucleus. After incubation, cells were washed four times with PBS, and intracellular MDC and LC3 were measured after the slides were mounted in Gelvatol. Confocal immunofluorescence analysis was performed with settings optimized for DAPI, TRITC and FITC.

For the endogenous protein assay, the cells were fixed and permeabilized. The samples were blocked in 2% goat serum for 1 h and incubated with primary antibodies at 4 °C overnight. The samples were then washed and incubated with the corresponding secondary antibodies. After three PBS washes, the cells were analyzed using confocal microscopy.

### Transmission electron microscopy

Cells were fixed with 4% glutaraldehyde and post-fixed in 1% osmium tetroxide at 4°C. The samples were then washed again, dehydrated with graded alcohol, and embedded in Epon-Araldite resin. Next, 50 nm of ultrathin sections were obtained using an ultramicrotome (Leica). Sections were then stained with uranyl acetate and lead citrate. A Hitachi H-7500 transmission electron microscope was used to observe autophagosomes.

### Protein stability assay, western blotting analyses and immunoprecipitation studies

GRP78 protein degradation was analyzed by CHX-chase analysis. HCT-116 cells were pre-incubated with or without 120 μM berberine for 24 h. Subsequently, 5 μg/ml CHX was added to inhibit protein synthesis. The cells were collected at 0, 2, 4, 6, 8 and 10 h following treatment with CHX.

Total protein in the supernatant was measured using the bicinchoninic acid (BCA) protein assay kit (P0010-1, Beyotime, China). Cells were lysed supplemented with protease inhibitors PMSF.

For the co-IP studies, the lysates were centrifuged for 15 min at 13,000× *g* and the resulting supernatant was precleared by incubation with Protein A (for rabbit antibody) or G (for mouse antibody) for 1 h at 4°C. The precleared supernatant was subjected to overnight immunoprecipitation using the indicated antibodies or control IgG antibodies at 4°C. On the next day, protein complexes were collected by incubation with Protein A (for rabbit antibody) or G (for mouse antibody) for 2 h at 4°C. The collected protein complexes were washed five times with co-immunoprecipitation buffer and eluted by boiling in protein sample buffer under reducing conditions. Sample proteins were then resolved by SDS-PAGE and analyzed by western blotting.

The lysates were boiled for 5 min and separated by 10–12% SDS-PAGE before the proteins were transferred onto PVDF membranes (Millipore). After blocking in 5% skimmed milk for 1 h, the membranes were rinsed and probed with 1:1000-diluted primary antibodies against LC3, GRP78, ATF6, and Ub overnight at 4°C, followed by horseradish peroxidase (HRP)-conjugated secondary antibodies for 2 h at room temperature. Next, the immunoreactive protein bands were visualized using the enhanced chemiluminescence detection kit (PerkinElmer, Waltham) and X-ray film. Finally, the blots were scanned, and densitometric analysis was performed on the scanned images using Image J Software.

### Construction, expression, purification of plasmid His-GRP78

The target gene GRP78 was subcloned into the pET-28a plasmid to generate the recombinant plasmid pET-28a-GRP78. The recombinant plasmid was then confirmed by restriction analysis using *BamHI* and *XhoI*, PCR and sequencing by Sangon Biotech Co., Ltd. Next, BL21 was transformed with pET-28a-GRP78, which was cultured at 37°C until the OD_600_ reached 0.8, and IPTG was added to a final concentration of 1mM to induce GRP78 expression.

BL21 cells expressing GRP78 were harvested by centrifugation, and resuspended in 10 mM phosphate buffered saline (PBS, pH7.4) for sonication on ice. After centrifugation, the supernatant was then collected and applied into a 10 ml Ni^2+^- Sepharose 6 Fast Flow column pre-equilibrated. The column was eluted with 50, 100, 150, 200, 250, 300, 400 and 500 mM imidazole. The eluted fraction of GRP78 protein was dialyzed overnight in 10 mM PBS to remove imidazole. The purity of GRP78 was analyzed using 10% SDS-PAGE.

### Fluorescence spectra measurements

Quantitative analysis of the potential interaction between berberine and GRP78 was performed by fluorometric titration. The GRP78 sample was purified and berberine was dissolved into double distilled water between 60-80°C. A 1.0 mL solution of GRP78 was added to 1.0 cm quartz cells at a given temperature and was titrated by successive addition of berberine. Berberine was mixed with GRP78 and incubated for 5 min to allow the reagents to react prior to the measurements. The fluorescence emission spectra were recorded in the range of 315–600 nm. Both slits of excitation and emission were 10 nm, with an excitation wavelength at 295 nm and an optical path of 10 mm.

### Statistical analysis

All experiments were performed in triplicate, and a representative experiment was selected for presentation. Data were expressed as the mean ± SEM. Differences among groups were tested by one-way analysis of variance (ANOVA) and a p-value <0.05 was considered statistically significant. Comparisons between two groups were evaluated using Student's t-test.

## SUPPLEMENTARY MATERIALS FIGURES AND TABLES



## Abbreviations

GRP78: Glucose-regulated protein 78
MTT: Methyl thiazolyl tetrazolium
SDS-PAGE: Sodium dodecyl sulfate polyacrylamide gel electrophoresis
PVDF: polyvinylidene fluoride
MDC: monodansylcadaverin
ATF6: activating transcription factor 6
YY1: Yin Yang 1
CQ: chloroquine
BAF: Bafilomycin
3-MA: 3-methyladenine
CHX: cycloheximide

## References

[1] Boya P, Reggiori F, Codogno P (2013). Emerging regulation and functions of autophagy. Nature cell biology.

[2] Vicinanza M, Korolchuk VI, Ashkenazi A, Puri C, Menzies FM, Clarke JH, Rubinsztein DC (2015). PI(5)P regulates autophagosome biogenesis. Molecular cell.

[3] Nixon RA (2013). The role of autophagy in neurodegenerative disease. Nature medicine.

[4] Nakatogawa H, Suzuki K, Kamada Y, Ohsumi Y (2009). Dynamics and diversity in autophagy mechanisms: lessons from yeast. Nature reviews Molecular cell biology.

[5] Wang Y, Huang C, Zhang H, Wu R (2015). Autophagy in glaucoma: Crosstalk with apoptosis and its implications. Brain research bulletin.

[6] Altman BJ, Rathmell JC (2012). Metabolic stress in autophagy and cell death pathways. Cold Spring Harbor perspectives in biology.

[7] Yang ZJ, Chee CE, Huang S, Sinicrope F (2011). Autophagy modulation for cancer therapy. Cancer biology & therapy.

[8] Wang N, Feng Y, Zhu M, Tsang CM, Man K, Tong Y, Tsao SW (2010). Berberine induces autophagic cell death and mitochondrial apoptosis in liver cancer cells: the cellular mechanism. Journal of cellular biochemistry.

[9] Scriven P, Coulson S, Haines R, Balasubramanian S, Cross S, Wyld L (2009). Activation and clinical significance of the unfolded protein response in breast cancer. British journal of cancer.

[10] Gomez BP, Riggins RB, Shajahan AN, Klimach U, Wang A, Crawford AC, Zhu Y, Zwart A, Wang M, Clarke R (2007). Human X-box binding protein-1 confers both estrogen independence and antiestrogen resistance in breast cancer cell lines. FASEB journal.

[11] Verfaillie T, Garg AD, Agostinis P (2013). Targeting ER stress induced apoptosis and inflammation in cancer. Cancer letters.

[12] Ni M, Zhang Y, Lee AS (2011). Beyond the endoplasmic reticulum: atypical GRP78 in cell viability, signalling and therapeutic targeting. The Biochemical journal.

[13] Ron D, Walter P (2007). Signal integration in the endoplasmic reticulum unfolded protein response. Nature reviews Molecular cell biology.

[14] Li J, Lee AS (2006). Stress induction of GRP78/BiP and its role in cancer. Current molecular medicine.

[15] Lee AS (2001). The glucose-regulated proteins: stress induction and clinical applications. Trends in biochemical sciences.

[16] Jagannathan S, Abdel-Malek MA, Malek E, Vad N, Latif T, Anderson KC, Driscoll JJ (2015). Pharmacologic screens reveal metformin that suppresses GRP78-dependent autophagy to enhance the anti-myeloma effect of bortezomib. Leukemia.

[17] Cook KL, Shajahan AN, Warri A, Jin L, Hilakivi-Clarke LA, Clarke R (2012). Glucose-regulated protein 78 controls cross-talk between apoptosis and autophagy to determine antiestrogen responsiveness. Cancer research.

[18] Cha-Molstad H, Sung KS, Hwang J, Kim KA, Yu JE, Yoo YD, Jang JM, Han DH, Molstad M, Kim JG, Lee YJ, Zakrzewska A, Kim SH (2015). Amino-terminal arginylation targets endoplasmic reticulum chaperone BiP for autophagy through p62 binding. Nature cell biology.

[19] Li J, Ni M, Lee B, Barron E, Hinton DR, Lee AS (2008). The unfolded protein response regulator GRP78/BiP is required for endoplasmic reticulum integrity and stress-induced autophagy in mammalian cells. Cell death and differentiation.

[20] Tang J, Feng Y, Tsao S, Wang N, Curtain R, Wang Y (2009). Berberine and Coptidis rhizoma as novel antineoplastic agents: a review of traditional use and biomedical investigations. Journal of ethnopharmacology.

[21] Tillhon M, Guaman Ortiz LM, Lombardi P, Scovassi AI (2012). Berberine: new perspectives for old remedies. Biochemical pharmacology.

[22] Derosa G, Maffioli P, Cicero AF (2012). Berberine on metabolic and cardiovascular risk factors: an analysis from preclinical evidences to clinical trials. Expert opinion on biological therapy.

[23] Inoue K, Kulsum U, Chowdhury SA, Fujisawa S, Ishihara M, Yokoe I, Sakagami H (2005). Tumor-specific cytotoxicity and apoptosis-inducing activity of berberines. Anticancer research.

[24] Lin JP, Yang JS, Lee JH, Hsieh WT, Chung JG (2006). Berberine induces cell cycle arrest and apoptosis in human gastric carcinoma SNU-5 cell line. World J Gastroenterol.

[25] Choi MS, Yuk DY, Oh JH, Jung HY, Han SB, Moon DC, Hong JT (2008). Berberine Inhibits Human Neuroblastoma Cell Growth through Induction of p53-dependent Apoptosis. Anticancer research.

[26] Ko (2009). Coptis chinensis inhibits hepatocellular carcinoma cell growth through nonsteroidal anti-inflammatory drug-activated gene activation. International Journal of Molecular Medicine.

[27] Mantena SK, Sharma SD, Katiyar SK (2006). Berberine inhibits growth, induces G1 arrest and apoptosis in human epidermoid carcinoma A431 cells by regulating Cdki-Cdk-cyclin cascade, disruption of mitochondrial membrane potential and cleavage of caspase 3 and PARP. Carcinogenesis.

[28] Tsang CM, Cheung KC, Cheung YC, Man K, Lui VW, Tsao SW, Feng Y (2015). Berberine suppresses Id-1 expression and inhibits the growth and development of lung metastases in hepatocellular carcinoma. Biochimica et biophysica acta.

[29] Peng PL, Kuo WH, Tseng HC, Chou FP (2008). Synergistic tumor-killing effect of radiation and berberine combined treatment in lung cancer: the contribution of autophagic cell death. International journal of radiation oncology, biology, physics.

[30] Ho YT, Lu CC, Yang JS, Chiang JH, Li TC, Ip SW, Hsia TC, Liao CL, Lin JG, Wood WG, Chung JG (2009). Berberine induced apoptosis via promoting the expression of caspase-8, -9 and -3, apoptosis-inducing factor and endonuclease G in SCC-4 human tongue squamous carcinoma cancer cells. Anticancer research.

[31] Hsu WH, Hsieh YS, Kuo HC, Teng CY, Huang HI, Wang CJ, Yang SF, Liou YS, Kuo WH (2007). Berberine induces apoptosis in SW620 human colonic carcinoma cells through generation of reactive oxygen species and activation of JNK/p38 MAPK and FasL. Archives of toxicology.

[32] Tang F, Wang D, Duan C, Huang D, Wu Y, Chen Y, Wang W, Xie C, Meng J, Wang L, Wu B, Liu S, Tian D (2009). Berberine inhibits metastasis of nasopharyngeal carcinoma 5-8F cells by targeting Rho kinase-mediated Ezrin phosphorylation at threonine 567. The Journal of biological chemistry.

[33] Fukuda K, Hibiya Y, Mutoh M, Koshiji M, Akao S, Fujiwara H (1999). Inhibition by berberine of cyclooxygenase-2 transcriptional activity in human colon cancer cells. Journal of ethnopharmacology.

[34] Chung JG, Chen GW, Hung CF, Lee JH, Ho CC, Ho HC, Chang HL, Lin WC, Lin JG (2000). Effects of berberine on arylamine N-acetyltransferase activity and 2-aminofluorene-DNA adduct formation in human leukemia cells. The American journal of Chinese medicine.

[35] Li Z, Wang Y, Newton IP, Zhang L, Ji P, Li Z (2015). GRP78 is implicated in the modulation of tumor aerobic glycolysis by promoting autophagic degradation of IKKbeta. Cellular signalling.

[36] Baumeister P, Luo S, Skarnes WC, Sui G, Seto E, Shi Y, Lee AS (2005). Endoplasmic reticulum stress induction of the Grp78/BiP promoter: activating mechanisms mediated by YY1 and its interactive chromatin modifiers. Mol Cell Biol.

[37] Zhang Y, Tseng CC, Tsai YL, Fu X, Schiff R, Lee AS (2013). Cancer cells resistant to therapy promote cell surface relocalization of GRP78 which complexes with PI3K and enhances PI(3,4,5)P3 production. PloS one.

[38] Chang Yi-Wen, Chen Hsin-An, Tseng Chi-Feng, Hong Chih-Chen, Jui-Ti Ma (2014). De-acetylation and degradation of HSPA5 is critical for E1A metastasis suppression in breast cancer cells. Oncotarget.

[39] Baehrecke EH (2005). Autophagy: dual roles in life and death?. Nature reviews Molecular cell biology.

[40] Jayanta Debnath EHB, Guido Kroemer (2005). Does Autophagy Contribute to Cell Death? Autophagy.

[41] Fan X, Wang J, Hou J, Lin C, Bensoussan A, Chang D, Liu J, Wang B (2015). Berberine alleviates ox-LDL induced inflammatory factors by up-regulation of autophagy via AMPK/mTOR signaling pathway. J Transl Med.

[42] Wang J, Qi Q, Feng Z, Zhang X, Huang B, Chen A, Prestegarden L, Li X, Wang J (2016). Berberine induces autophagy in glioblastoma by targeting the AMPK/mTOR/ULK1-pathway. Oncotarget.

[43] Lee YS, Kim WS, Kim KH, Yoon MJ, Cho HJ, Shen Y, Ye JM, Lee CH, Oh WK, Kim CT, Hohnen-Behrens C, Gosby A, Kraegen EW (2006). Berberine, a natural plant product, activates AMP-activated protein kinase with beneficial metabolic effects in diabetic and insulin-resistant states. Diabetes.

