# Supplementary material for: Berberine-induced autophagic cell death by elevating GRP78 levels in cancer cells
Source: Oncotarget. 2017 Feb 1;8(13):20909–24. doi: 10.18632/oncotarget.14959 (PMC5400555; doi:10.18632/oncotarget.14959)
Supplement: Supplementary file 1 [file oncotarget-08-20909-s001.pdf]

Berberine-induced autophagic cell death by elevating GRP78 levels in cancer cells

SUPPLEMENTARY FIGURES AND TABLES

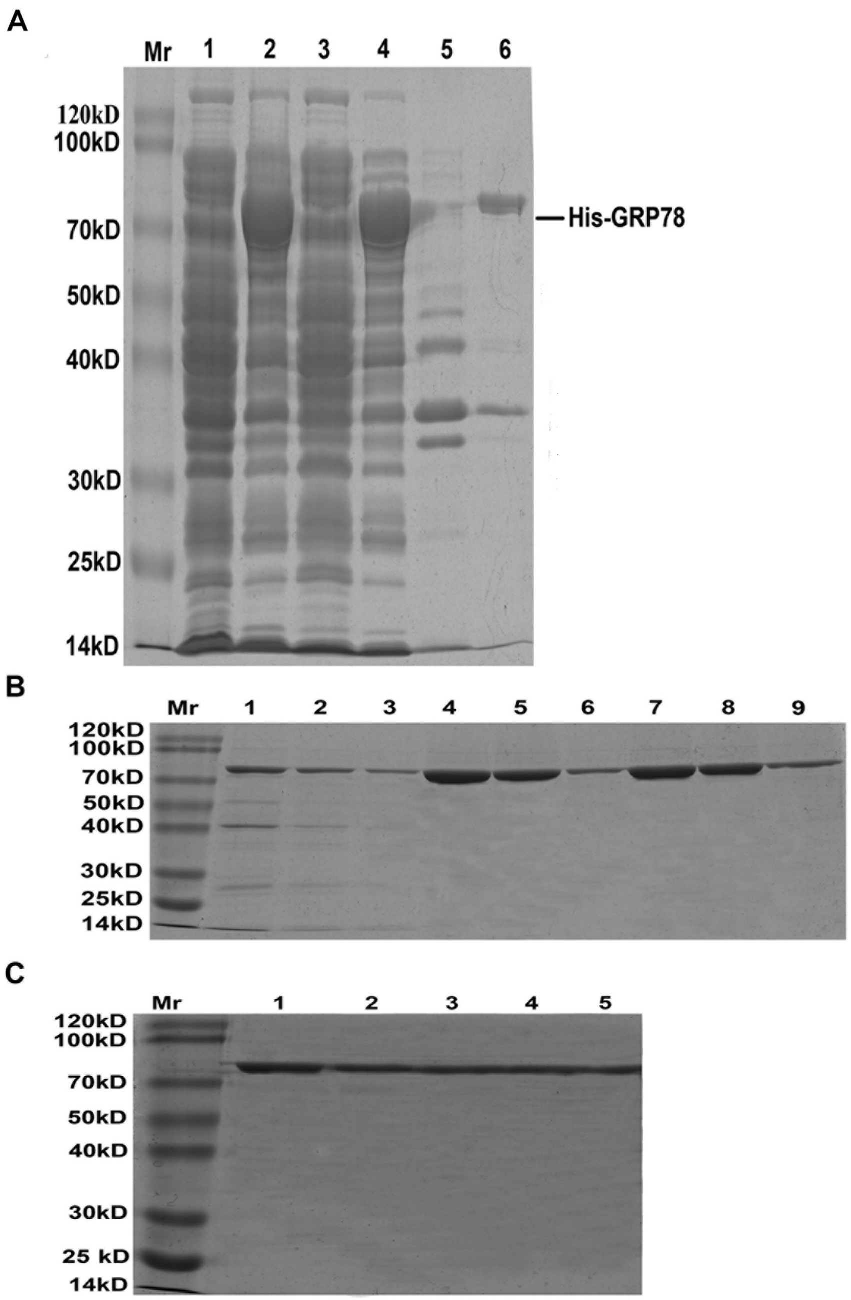

Supplementary Figure 1: Expression, purification of plasmid His-GRP78 and binding constant, binding sites between berberine and GRP78 A. Lane Mr, Protanein marker; Lanes 1, 3, 5 and 2, 4, 6 showed protein levels in the cell lysate, supernatant and pellet fractions from BL21 expressing His-GRP78 at 37°C in the absence or presence of 1 mM IPTG. **B.** Analysis of purified His-GRP78 by SDS-PAGE gel. Lane Mr, Protein marker; Lanes 1-3, 4-6, 7-9 showed that His-GRP78 was eluted with 50 mM,100 mM and 150 mM imidazole, respectively. **C.** Lane Mr, Protein marker; Lanes 1-5 showed that His-GRP78 was eluted with 200 mM, 250 mM, 300 mM, 400 mM and 500 mM imidazole, respectively.

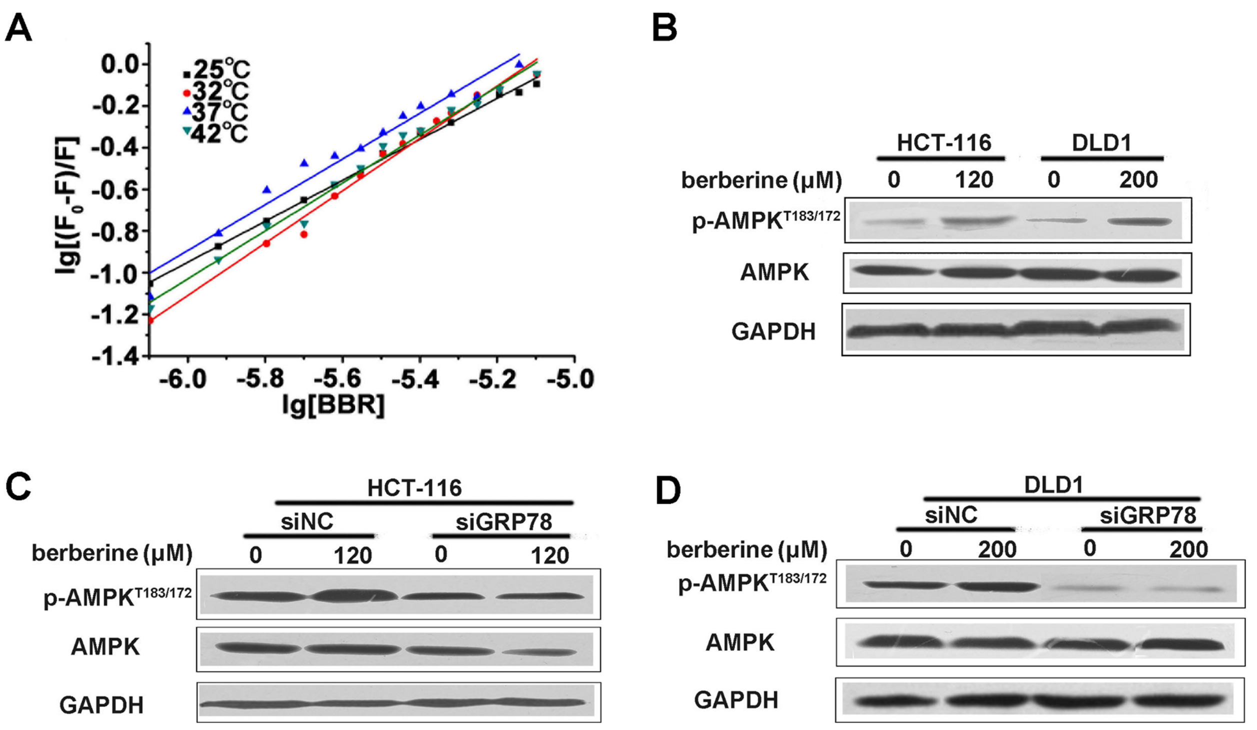

Supplementary Figure 2: The binding constant, binding sites between berberine and GRP78 and berberine activated p-AMPK$^{T183/172}$ by GRP78 A. The values of K_A, n, and R^2 were determined with the equation: $lg(F_0\text{-}F/F) = lg\ K_A + n\ lg[\ berberine\ ]_f$ by linear regression of plots of lg $(F_0\text{-}F)/F$ vs lg$[berberine]_f$ at different temperature. **B.** Western blot of AMPK and p-AMPK$^{T183/172}$ were performed on lysates from HCT-116 and HepG2 cells that were treated with berberine at the indicated concentrations for 24 h. **C** and **D.** HCT-116 (C) and DLD1(D) cells were transfected with control siRNA or siRNA targeting GRP78, respectively. After 48 h, the cells were treated with indicated concentrations of berberine for 24 h, AMPK and p-AMPK$^{T183/172}$ were measured by western blot assays.

Supplementary Table 1: Primers used for real-time PCR

Gene	Forward Primer (5′-3′)	Reverse Primer (5′-3′)
GRP78	CTGTGCAGCAGGACATCAAGTTC	TGTTTGCCCACCTCCAATATCA
LC3-II	AGAAGGCGCTTACAGCT	TCCTGGGAGGCATAGAC
ATF6	GAATCCGCTTGTCAGTCTCG	CCGCTTCAGTGTTCCATTTT
GAPDH	GCACCGTCAAGGCTGAGAAC	TGGTGAAGACGCCAGTGGA

The primers used for real-time PCR in our study.

Supplementary Table 2: Values of K_A and n of the interaction between berberine and GRP78

T (°C)	$K_A/10^5$ ($L \cdot mol^{-1}$)	n	R^2
25	6.433	1.257	0.9852
32	5.880	1.152	0.9852
37	5.334	1.032	0.9691
42	4.949	0.983	0.9955

The values of K_A, n and R^2, which were determined with the equation: lg(F_0-F/F)=lg K_A +n lg[berberine $]_f$ by linear regression of plots of lg (F_0-F)/F vs lg[berberine$]_f$ at different temperature.

Supplementary Table 3: Thermodynamic paramenters of interaction between berberine and GRP78

T (°C)	ΔG ($KJ \cdot mol^{-1}$)	ΔH ($KJ \cdot mol^{-1}$)	ΔS ($J \cdot mol^{-1} \cdot K^{-1}$)
25	-33.266	-31.536	-0.070
32	-33.815		
37	-34.116		
42	-34.467		

The thermodynamic parameters dependent on temperatures were calculated by the following thermodynamic equations:

$\Delta G=\Delta H-T\Delta S$ (1)

ΔG=-RT lnK (2)

$\ln (k_2/k_1) = (1/T_1-1/T_2)\Delta H/R$ (3)

Where ΔG represented the free energy change, R = 8.314472 J/mol K was the gas constant.
